# Editorial: Microbial modulation to mitigate the impact of climate change on wine production

**DOI:** 10.3389/fmicb.2024.1465637

**Published:** 2024-08-07

**Authors:** Alice Vilela, Paola Domizio, Antonio Morata

**Affiliations:** ^1^Chemistry Research Centre–Vila Real (CQ-VR), Department of Agronomy, School of Agrarian and VeterinarySciences (ECAV), University of Trás-os-Montes and Alto Douro, Vila Real, Portugal; ^2^DAGRI-Department of Agriculture, Food, Environment and Forestry, University of Florence, Florence, Italy; ^3^EnotecUPM, ETSIAAB, Universidad Politécnica de Madrid, Madrid, Spain

**Keywords:** non-saccharomyces yeasts, mixed-culture fermentations on wine quality, biotechnological strategies to improve wine production and safety, bioprotection and sulfur dioxide reduction, non-thermal technologies

Microorganisms in vineyards and the surrounding soil can alter the composition of the final wine. The microbial community changes at the beginning of the winemaking process, and different types of wine yeasts dominate the grape juice and wine environment. Weather extremes related to climate change can disrupt the microbial balance in the wine, leading to undesirable characteristics in the final product.

As winegrowers, winemakers, and scientists, your work is vital in preserving the quality of wine, especially in the face of climate change. The decrease in suitable viticulture areas and changes in grape composition present challenges. Many of you are studying yeasts and bacteria to mitigate these issues in warmer climates. Your work is significant and essential for improving wine quality through understanding and managing microorganisms in the vineyard and during winemaking.

As winegrowers, winemakers, and scientists, you are not just at the forefront of mitigating the risks of climate change in the wine industry; you are also shaping its future. Recent advancements in 'omics' technologies have provided new opportunities for us to understand the grape/wine microbial ecosystem better. Specifically, unconventional non-*Saccharomyces* species, previously considered as spoilage microorganisms, are now recognized as beneficial as they enhance the wine aroma and taste when cultivated in controlled fermentations with *Saccharomyces cerevisiae*. Furthermore, ongoing biological approaches for modifying wine acidity using *Saccharomyces* and non-*Saccharomyces* yeasts and traditional lactic acid bacteria such as *Oenococcus oeni* and *Lactiplantibacillus plantarum* are being explored. This Research Topic explores how climate change can impact microbial diversity and subsequently alter wine characteristics. These risks can be mitigated by regulating the microbial community and utilizing yeast derivatives to enhance wine aroma and taste. Your work is not just important; it is empowering, as you are responsible for shaping the future of winemaking.

This Research Topic comprises six types of work—one mini-review article, one review article, and four original research articles—written by international researchers to provide up-to-date research on the different dimensions of the vast research world of Microbial Modulation to Mitigate the Impact of Climate Change on Wine Production.

The mini-review article (Comuzzo et al.) delves into emerging biotechnologies and non-thermal technologies for winemaking in the context of global warming. According to the authors, using non-*Saccharomyces* yeast species, such as *Lachancea thermotolerans*, can help manage some global warming wine issues by stabilizing pH and reducing alcohol content.

Lower-pH wine improves freshness, palatability, and microbiological stability. Certain yeast species, like *Hanseniaspora* spp. and *Metschnikowia pulcherrima*, can enhance aroma complexity, improve the wine's sensory profile, and aid in acidification and bio-protection in winemaking.

Bio-protection helps control oxidation, inhibit wild microorganisms, improve the implantation of starters, and limit SO_2_. Reductive yeast derivatives with high contents of reducing peptides and compounds like glutathione are also helpful in reducing SO_2_ content. Also, emerging non-thermal technologies like Ultra High-Pressure Homogenization (UHPH) and Pulsed Light (PL) improve wine stability by controlling microbes and deactivating oxidative enzymes, which enhances the use of emerging non-*Saccharomyces* and reduces SO_2_ additions (Comuzzo et al.).

Puyo et al. studied the topic of bio-protection in enology by *M. pulcherrima*: From field results to a scientific inquiry. They wrote an interesting mini-review article, which mainly focuses on bio-protection using a non-*Saccharomyces* yeast, *M. pulcherrima*, recommended for bio-protecting grape musts. As they state, there are still many unanswered questions about the production of toxic compounds by *M. pulcherrima*, such as its potential production of killer toxins and its ability to make cell-cell contact interactions. The authors also point out that *M. pulcherrima* is known for consuming much oxygen. This high consumption could quickly deplete the oxygen in the grape must, preventing it from entering the pathways that lead to wine browning and producing unwanted aromas. Furthermore, *M. pulcherrima* secretes pulcherriminic acid, which chelates Fe^3+^ once released into the medium. This ion is involved in redox mechanisms through the Fenton reaction. Reducing iron in the medium by pulcherriminic acid may also help, to a lesser extent, protect the grape-must from oxidation. This brief review examines the current state of field trials and laboratory studies demonstrating the effects of using yeasts for bio-protection and the interaction mechanisms responsible for these effects.

*M. pulcherrima* was also the focus of the research article published by Torrellas et al.. The study delved into using non-*Saccharomyces* yeasts as starters in winemaking, namely the industrial problem of efficiently propagating this type of yeast. The work shows that the poor growth of *Hanseniaspora vineae* and *M. pulcherrima* in molasses is related to deficient sucrose consumption and low invertase activity.

The authors modified the cultivation media to address this Research Topic, which involved hydrolysis and reducing the sucrose concentration. The results indicated that both species showed improved biomass production when cultivated in a hexose-based media, effectively addressing their low invertase activity. Reducing the sugar concentration also led to a respiratory metabolism, resulting in a higher biomass yield.

However, the modifications did not enhance biomass production due to reduced sugar availability. To assess the effectiveness of these changes, fermentations using mixed grape juice with biomass produced under conditions similar to *M. pulcherrima* and *S. cerevisiae* were conducted. The analysis of the resulting wines indicated that the treatments tested did not negatively impact wine quality, demonstrating their potential for practical application on an industrial scale to improve biomass production.

In an original research article, Vion et al. explored the intracellular metabolic variations between seventeen *S. cerevisiae* strains belonging to two different genetic populations, flor yeasts and wine yeasts, in alcoholic fermentation. These two populations are closely related, share the same ecological niche, and have distinct genetic characteristics. The authors developed a 1H-NMR protocol to measure the intracellular concentration of yeast biomass during the alcoholic fermentation of natural grape juice. The protocol was used to analyze the different metabolic contents of several *S. cerevisiae* strains (flor and wine yeasts). Additionally, accurate quantification of 21 metabolites in a two-time series provided new results that showed that intracellular metabolic variability is influenced by the sampling time and the yeast strain, with complex interactions that prevent simple physiological conclusions.

Dournes et al. in an original research work, focused on the question of copper's impact on varietal thiols in wine, taking into account the use of this compound in organic vineyard management as the sole fungal control pesticide against downy mildew. Colombard and Gros Manseng grape juices were fermented under different copper levels to mimic the consequences in grape-must of organic practices. The authors found that the highest copper level for both grape varieties significantly increased yeast consumption of precursors. Also, for both grape varieties, free thiol content in wine significantly decreased.

The amount of thiol produced during fermentation remained constant for Colombard grapes, regardless of copper levels, indicating that copper only had an oxidative effect on this variety. However, during Gros Manseng fermentation, the thiol content increased as the copper content increased, showing a potential 90% increase. This suggests that copper might alter the pathways for producing varietal thiols, highlighting the significant role of oxidation. These findings contribute to understanding how copper affects thiol-focused fermentation and emphasize the importance of considering total thiol production (both reduced and oxidized) to comprehend the impact of studied factors better and distinguish between chemical and biological effects.

Considering the importance of yeast co-inoculations in winemaking, which aims to modulate the aromatic profiles of wines, Bordet et al. investigated the impact of three co-cultures and corresponding pure cultures of *S. cerevisiae* on the chemical composition and sensory profile of Chardonnay wine.

The use of co-cultures affected esters, fatty acids, and phenol families. Comparisons between the sensory profiles and metabolomes of co-cultures, pure cultures, and associated wine blends have revealed distinct differences. It was observed that the co-culture did not simply combine the characteristics of the two pure-culture wines, indicating an interaction effect. High-resolution mass spectrometry identified thousands of biomarkers specific to the co-cultures. Additionally, the study highlighted the involvement of metabolic pathways, particularly those related to nitrogen metabolism, in the observed changes in wine composition.

The authors concluded that mixed *S. cerevisiae* yeasts can modulate the aromatic and chemical profile of wines without affecting their fermentative properties. They found that traditional methods, like monitoring yeast populations over time, are insufficient for understanding the interactions between yeast strains. A comprehensive approach involving different techniques is necessary to comprehend these interactions fully.

In summary, the Research Topic “*Microbial modulation to mitigate the impact of climate change on wine production*” demonstrates that microbial modulation can mitigate the impact of climate change on wine production using different techniques, yeast strains, and biochemical approaches. Moreover, when conjugated with cutting-edge analytical methods, biotechnologies, and non-thermal technologies, it helps improve wine quality and safety.

## Author contributions

AV: Writing – original draft, Writing – review & editing, Conceptualization, Project administration. PD: Writing – review & editing, Conceptualization, Project administration, Validation. AM: Writing – review & editing, Conceptualization, Project administration, Validation.

